# Sexual harassment, sexual assault and rape by colleagues in the surgical workforce, and how women and men are living different realities: observational study using NHS population-derived weights

**DOI:** 10.1093/bjs/znad242

**Published:** 2023-09-12

**Authors:** Christopher T Begeny, Homa Arshad, Tamzin Cuming, Daljit K Dhariwal, Rebecca A Fisher, Marieta D Franklin, Philippa C Jackson, Greta M McLachlan, Rosalind H Searle, Carrie Newlands

**Affiliations:** Faculty of Health and Life Sciences, Department of Psychology, University of Exeter, Exeter, UK; Barts Bone and Joint Health, Barts NHS Trust, Royal London Hospital, London, UK; Department of Surgery, Homerton University Hospital, London, UK; Oxford University Hospitals NHS Foundation Trust, Nuffield Department of Surgical Sciences, University of Oxford, John Radcliffe Hospital, Oxford, UK; School of Medical Sciences, Division of Medical Education, University of Manchester, Manchester, UK; Department of Trauma and Orthopedics, Liverpool University Hospitals NHS Foundation Trust, Liverpool, UK; Department of Surgery, North Bristol NHS Trust, Bristol, UK; Department of Surgery, Frimley Health Foundation Trust, Frimley, UK; Adam Smith Business School, University of Glasgow, Glasgow, UK; School of Biosciences and Medicine, University of Surrey, Guildford, UK

## Abstract

**Background:**

This observational study, paired with National Health Service (NHS) workforce population data, examined gender differences in surgical workforce members’ experiences with sexual misconduct (sexual harassment, sexual assault, rape) among colleagues in the past 5 years, and their views of the adequacy of accountable organizations in dealing with this issue.

**Methods:**

This was a survey of UK surgical workforce members, recruited via surgical organizations.

**Results:**

Some 1704 individuals participated, with 1434 (51.5 per cent women) eligible for primary unweighted analyses. Weighted analyses, grounded in NHS England surgical workforce population data, used 756 NHS England participants. Weighted and unweighted analyses showed that, compared with men, women were significantly more likely to report witnessing, and be a target of, sexual misconduct. Among women, 63.3 per cent reported being the target of sexual harassment *versus* 23.7 per cent of men (89.5 per cent witnessing *versus* 81.0 per cent of men). Additionally, 29.9 per cent of women had been sexually assaulted *versus* 6.9 per cent of men (35.9 per cent witnessing *versus* 17.1 per cent of men), with 10.9 per cent of women experiencing forced physical contact for career opportunities (a form of sexual assault) *versus* 0.7 per cent of men. Being raped by a colleague was reported by 0.8 per cent of women *versus* 0.1 per cent of men (1.9 per cent witnessing *versus* 0.6 per cent of men). Evaluations of organizations’ adequacy in handling sexual misconduct were significantly lower among women than men, ranging from a low of 15.1 per cent for the General Medical Council to a high of 31.1 per cent for the Royal Colleges (men’s evaluations: 48.6 and 60.2 per cent respectively).

**Conclusion:**

Sexual misconduct in the past 5 years has been experienced widely, with women affected disproportionately. Accountable organizations are not regarded as dealing adequately with this issue.

## Teaser

This observational study paired with National Health Service (NHS) workforce population data examined gender differences in surgical workforce members’ experiences with sexual misconduct among colleagues over the past 5 years, and their views of the adequacy of the General Medical Council (GMC), NHS Trusts and other accountable organizations’ handling of this issue. Results of weighted and unweighted analyses showed that women and men in the surgical workforce are living different realities—women are both experiencing and witnessing sexual misconduct more often than men. Women also have significantly lower evaluations of organizations’ handling of sexual misconduct, ranging from a low of 15.1 per cent for the GMC to a high of 31.1 per cent for the Royal Colleges.

## Introduction

Sexual harassment, sexual assault, and rape, referred to as sexual misconduct, are unacceptable. Sexual misconduct occurs frequently and appears to go unchecked in the surgical environment owing to a combination of a deeply hierarchical structure and a gender and power imbalance. Sexual misconduct in surgery is not new, and sexual misconduct in wider healthcare is a global issue. Evidence from the UK, USA, Australia, and many other countries indicates that this is also a significant risk to patient safety^1,2^.

In 2021, sexual misconduct in surgery in the UK was called out by two trainees^3^, which led women surgeons to share their own workplace experiences^4,5^. Mainstream media attention and statements from a variety of leaders in UK surgery and healthcare followed^6,7^. Although there are some small-scale surveys^8,9^ on sexual misconduct in focal settings , and others^10^ on sexism defined more broadly, the extent of sexual misconduct perpetrated by colleagues within the UK surgical workforce has not been reported to date.

There are reasons for the paucity of data, including under-reporting of sexual misconduct, fear of potential repercussions, and damage to career progression^11^. Following Fleming and Fisher’s article^3^, websites such as Project S^12^ and Surviving In Scrubs^13^ were set up, collating healthcare workers’ experiences of being subjected to sexism, misogyny, and sexual misconduct.

In 2022, the Working Party on Sexual Misconduct in Surgery (WPSMS)^14^ was established, with the aims of gathering data and promoting sexual safety in the surgical working environment. To facilitate organizational and cultural change, quantitative data were collected on workforce members’ experiences with sexual misconduct using an ethically approved anonymous survey. The survey specifically examined sexual misconduct perpetrated by co-workers (not patients). Such evidence regarding the behaviour of co-workers may be particularly actionable for accountable organizations. WPSMS devised this study, supported by multiple stakeholders with power to make working conditions safer for staff, and surgery safer for patients. Key findings from the study are reported here. Additional information is available in the *supplementary material*.

## Methods

This study had HRA/HCRW approval (Health Research Authority/Health and Care Research Wales; HRA reference 22/HRA/3738) and ethical clearance from the University of Exeter’s Faculty of Health and Life Sciences Psychology Research Ethics Committee (REC reference 511842).

### Patient and public involvement

Items in this survey were adapted based on feedback from people who had witnessed and/or been targets of sexual misconduct, including members of the UK healthcare workforce.

### Data collection

Following ethical approval, including of General Data Protection Regulation-compliant protocols, participants were invited to complete an online survey built using Qualtrics^©^ (Qualtrics, Provo, UT, USA). They were provided an information sheet, consent form, and debriefing page. Participants were signposted to sources of support at the beginning and end of the study, and a trigger warning preceded questions on sexual misconduct. To help mitigate selection bias and capture the widest possible sample, those invited were encouraged to participate regardless of whether they had or had not witnessed, or been targets of, sexual misconduct.

Participants were recruited via electronic lists held and circulated by relevant professional groups including surgical and anaesthetic Royal Colleges, Health Education England (HEE), and others (*supplementary material*). Participation was voluntary and not remunerated. Primary unweighted analyses included women and men aged at least 18 years who were on a standard UK medical workforce grade (foundation year 1/2–consultant) and responded to the key measures being analysed (for example on sexual misconduct; *supplementary material*). Weighted analyses, grounded in the National Health Service (NHS) England surgical workforce population data^15^, included a subset of participants who worked at NHS-based organizations in England (within subspecialties for which population data were available). To help maintain anonymity, tracking of Internet Protocol addresses was disabled. When recruitment extended to include relevant online social networks/sites (for example via accounts maintained by professional surgical groups), additional safeguards were built into the survey to prevent multiple submissions. The survey was open from September to December 2022.

### Measures

Items assessing sexual misconduct were adapted from previous research^16,17^, modified based on feedback from healthcare professionals, including those affected, to ensure relevance and fit to this professional context. Participants were given structured guidelines for responding, including definitions of sexual harassment, sexual assault, and rape (*supplementary material*).

Sixteen items assessed participants’ experiences with witnessing sexual misconduct among colleagues. An analogous set assessed experiences of being a target. For each, participants were asked, ‘In the past 5 years (in any work-related context), how often have you witnessed, overheard or been present for [been the target or victim/survivor of]…[item]?’. Responses were given on a scale from 1 (never) to 5 (very often—a few times a week, or more). *Tables S1* and *S2* provide additional information and verbatim wording.

Participants were also asked to evaluate whether they believed relevant accountable organizations, including the General Medical Council (GMC), NHS Trusts, HEE, the British Medical Association (BMA), and the Royal Colleges, were addressing issues of sexual misconduct adequately. Responses were given on a scale from 1 (no, absolutely not) to 7 (yes, absolutely*)*, with an additional response option N/A (don’t know, or not applicable), which was excluded from the analyses. Key demographic questions included participant age, gender, current specialty, and grade.

### Statistical analyses

Primary analyses involved multivariable analyses of co-variance to test for mean gender differences. Additional analyses using linear regression tested whether individuals’ evaluations of the GMC, NHS Trusts, and other accountable organizations were further explained by the frequency at which they witnessed and/or were targets of sexual misconduct. All analyses were conducted in SPSS^®^ version 28 (IBM, Armonk, NY, USA) with participant age and grade as co-variates (complete-case analysis). Follow-up analyses also assessed the robustness of findings using additional co-variates, bootstrapping and other non-parametric tests, and multiple imputation. In addition, weighted analyses were conducted. These used population data for the NHS surgical workforce in England^15^ to generate case weights for participants from this population (additional information is available in *supplementary material*). This produced a sample for analyses that mirrored the true representation of women and men in the surgical workforce by grade and key subspecialties, and most directly illustrated issues of sexual misconduct within the NHS England workforce.

## Results

### Participants

Of 1704 participants, 1434 provided sufficient data to be included in primary unweighted analyses. *Table 1* provides additional information on participants. The two largest surgical subspecialties in the surgical workforce^15^ were also the largest represented in this sample: trauma and orthopaedic surgery (31.8 per cent) and general surgery (18.5 per cent). Similarly, the two largest grades in surgery^15^ were the largest represented in this sample: consultants (63.1 per cent) and specialty trainees (20.2 per cent). Weighted analyses included the 756 participants in the NHS England surgical workforce.

**Table 1:** Participant demographic information (unweighted proportions)

	No. of participants (%)
Gender ratio (women : men)*	738 : 696
**Age (years)**	
≤ 30†	142 (9.9)
31–35	222 (15.5)
36–40	200 (13.9)
41–45	185 (12.9)
46–50	178 (12.4)
51–55	178 (12.4)
56–60	143 (10.0)
61–65	93 (6.5)
66–70	55 (3.8)
≥ 70	38 (2.6)
**Grade**	
Consultant (doctor)	905 (63.1)
Trust grade registrar/SAS/specialty doctor	81 (5.6)
Post-CCT fellow	40 (2.8)
Specialty trainee	289 (20.2)
Core trainee	52 (3.6)
Trust grade SHO/clinical fellow	39 (2.7)
Foundation year 1/2	28 (2.0)
**Subspecialty**	
Trauma and orthopaedic surgery	456 (31.8)
General surgery	265 (18.5)
Anaesthetics	151 (10.5)
Plastic surgery	116 (8.1)
Oral and maxillofacial surgery	87 (6.1)
Otolaryngology	63 (4.4)
Urology	48 (3.3)
Vascular surgery	45 (3.1)
Neurosurgery	31 (2.2)
Cardiothoracic surgery	27 (1.9)
Other subspecialties†	145 (10.1)

The data are those for 1434 participants eligible for primary unweighted analyses that included individuals on doctor grades (additional information is available in *supplementary material*). This preserved the linear interpretability of grade as a co-variate without drawing assumed equivalences across doctor, dental, and nursing (or other) grades. Follow-up analyses without co-variates and thus inclusion of respondents across all grades (doctor, dental, nursing, or other) yielded very similar results. *The number of non-binary participants in this sample was relatively small and so for ethical reasons (preservation of anonymity) with concomitant statistical limitations, analyses focally compared women and men. †In line with approved research ethics protocols, to help preserve participant anonymity, smaller demographic subgroups have been aggregated in *Table 1* to maintain at least 25 participants in each row. SAS, specialty doctor and specialist grade; CCT, Certificate of Completion of Training; SHO, senior house officer.

### Witnessing sexual harassment, sexual assault, and rape among colleagues in the past 5 years

**Fig. 1:**
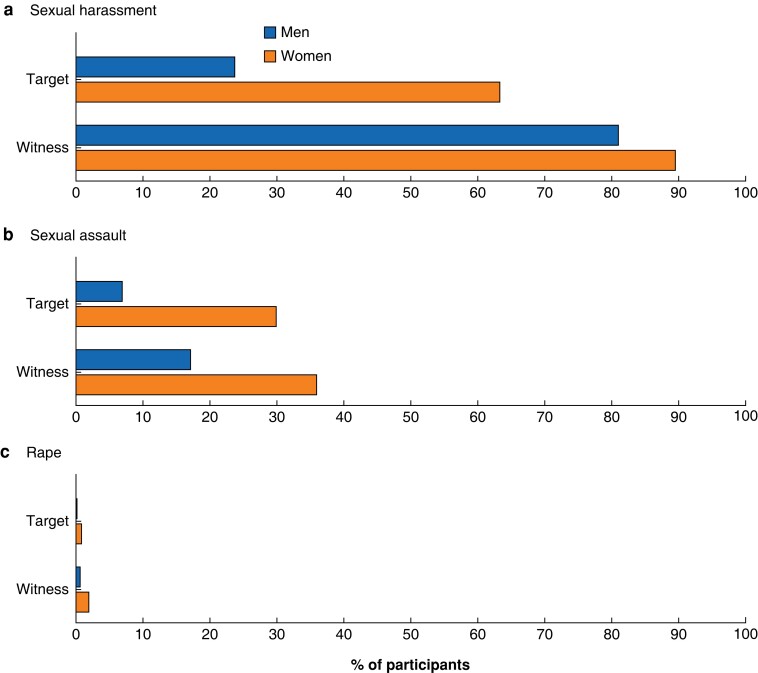
Percentage of participants who experienced witnessing, or being the target of, sexual harassment, sexual assault, and rape by gender in the past 5 years (unweighted) **a** Sexual harassment, **b** sexual assault, and **c** rape.

*Table 2* and *Fig. 1* show the overall proportions of women and men who had witnessed sexual harassment, sexual assault, and rape in the past 5 years (once or more). Mean frequencies are also provided, for both unweighted and weighted samples, with corresponding tests of gender differences.

**Table 2:** **Frequency of witnessing sexual harassment, sexual assault, and rape among colleagues in the past 5 years: differences between women and men controlling for age, grade (unweighted and weighted analyses)** *

	Witnessed sexual harassment, sexual assault, and rape among colleagues						
	Women		Men		Difference between genders		
	Yes (%)†	Frequency‡	Yes (%)†	Frequency‡	Difference in frequency§¶	*P*	*d*
**Harassment: composite**	89.5	1.69(0.57)	81.0	1.43(0.58)	0.26 (0.19, 0.32)	< 0.001	0.42
Jokes with sexual content	89.0	2.92(1.12)	80.6	2.76(1.12)	0.16 (0.04, 0.28)	0.012	0.14
Displaying sexualized pictures	29.7	1.45(0.77)	19.9	1.32(0.78)	0.13 (0.05, 0.22)	0.003	0.16
E-comms, unwanted/sexual	26.2	1.43(0.80)	16.1	1.29(0.81)	0.15 (0.06, 0.23)	0.001	0.17
Physical advances, unwanted/sexual	38.4	1.58(0.80)	14.9	1.26(0.81)	0.32 (0.23, 0.41)	< 0.001	0.38
Unwanted/sexual talk	61.8	2.14(1.06)	29.5	1.53(1.07)	0.60 (0.49, 0.72)	< 0.001	0.54
Uninvited comments about body	67.3	2.22(1.06)	38.3	1.68(1.07)	0.54 (0.42, 0.66)	< 0.001	0.48
Ask for a date despite previous refusal	18.0	1.23(0.53)	6.0	1.11(0.52)	0.12 (0.06, 0.18)	< 0.001	0.22
Offered career opportunities for sex	8.5	1.13(0.45)	2.7	1.05(0.44)	0.07 (0.02, 0.12)	0.004	0.16
Threatened for refusing sexual favour	5.5	1.08(0.35)	1.2	1.02(0.34)	0.06 (0.02, 0.10)	0.002	0.17
Deliberately infringing body space	44.9	1.84(1.01)	17.8	1.32(1.02)	0.52 (0.41, 0.63)	< 0.001	0.49
**Assault: composite**	35.9	1.23(0.40)	17.1	1.10(0.39)	0.13 (0.08, 0.17)	< 0.001	0.30
Forced contact for career opportunities.	16.6	1.26(0.59)	2.9	1.07(0.60)	0.19 (0.13, 0.26)	< 0.001	0.31
Touching, excluding genitals/breasts	33.2	1.53(0.86)	16.6	1.29(0.84)	0.24 (0.14, 0.33)	< 0.001	0.26
Touching of genitals/breasts	6.5	1.10(0.38)	1.6	1.03(0.39)	0.07 (0.02, 0.11)	0.002	0.17
Self-fondling by perpetrator	1.3	1.02(0.16)	0.3	1.01(0.18)	0.01 (−0.01, 0.03)	0.278	0.06
**Rape: composite**	1.9	1.01(0.13)	0.6	1.01(0.13)	0.01 (−0.01, 0.02)	0.453	0.04
Rape, workplace	0.6	1.01(0.13)	0.3	1.01(0.13)	0.00 (−0.01, 0.01)	0.968	0.00
Rape, other work contexts	2.0	1.02(0.16)	0.6	1.01(0.16)	0.01 (−0.01, 0.03)	0.221	0.06
**Weighted analyses**							
Harassment: composite	89.8	1.68(0.56)	84.2	1.44(0.55)	0.24 (0.15, 0.33)	< 0.001	0.38
Assault: composite	39.1	1.25(0.42)	21.7	1.15(0.42)	0.10 (0.03, 0.17)	0.004	0.21
Rape: composite	3.8	1.01(0.09)	2.5	1.02(0.09)	−0.01 (−0.02, 0.01)	0.408	0.06

Follow-up analyses testing the robustness of findings (for example with additional co-variates, non-parametric tests) yielded very similar results (also see †,¶). Additionally, as shown, weighted analyses yielded consistent results. †Proportion of women or men who witnessed this form of misconduct once or more in the past 5 years. These data provide an easily interpreted, descriptive illustration of the data. They are not integral to primary analyses, although follow-up analyses using this simplified (dichotomized, yes/no) form of the data yielded results that were highly consistent with those of primary analyses (for example, harassment composite, χ^2^(1) = 20.36, *P* < 0.001; assault composite, χ^2^(1) = 63.80, *P* < 0.001). ‡Mean(s.d.) frequency of witnessing each form of sexual misconduct (range of possible values 1–5). §Gender difference in mean frequency (95% c.i.), shown with *P* value and effect size (*d*) (general guide: *d* = 0.20, small effect; *d* = 0.50, medium effect)^18^. Overall, women and men differed in how often they witnessed sexual misconduct (across harassment, assault, and rape composites): multivariate F(3, 1410) = 22.67, *P* < 0.001, *d* = 0.44 (weighted: F(3, 727) = 13.98, *P* < 0.001, *d* = 0.48). Univariate tests indicated that women witnessed sexual harassment, and sexual assault, more often than men (harassment, F(1, 1412) = 63.89, *P* < 0.001, *d* = 0.42; assault, F(1, 1412) = 32.31, *P* < 0.001, *d* = 0.30; weighted: harassment, F(1, 729) = 26.37, *P* < 0.001, *d* = 0.38; assault, F(1, 729) = 8.48, *P* = 0.004, *d* = 0.21) (*supplementary material*). ¶Based on means estimated at the mean of co-variates (age, grade). Where significant (*supplementary material*), older ages and grades predicted lower frequencies (for example, harassment (displaying sexualized pictures), grade–frequency associated positively). Analyses included only respondents on doctor grades. This preserved the linear interpretability of this co-variate without drawing assumed equivalences across doctor, dental, and nursing (or other) grades. Follow-up analyses without co-variates and thus inclusion of respondents across all grades (doctor, dental, nursing, or other) yielded very similar results.

#### Unweighted analyses

Overall, women and men differed in how often they witnessed sexual misconduct. Univariate tests showed that women witnessed sexual harassment, and sexual assault, more often than men (*Table 2*).

#### Weighted analyses for NHS England

Results of weighted analyses provided further evidence that women witnessed sexual harassment, and sexual assault, more often than men (*Table 2*), even when examined in a sample that mirrored the true representation of women and men in the NHS England surgical workforce population.

### Being a target of sexual harassment, sexual assault, and rape among colleagues in the past 5 years

*Table 3* and *Fig. 1* provide the overall proportions of women and men who experienced being a target of sexual harassment, sexual assault, and rape in the past 5 years (once or more). Mean frequencies are also provided, for both unweighted and weighted samples, with corresponding tests of gender differences.

**Table 3:** **Frequency of being the target of sexual harassment, sexual assault, and rape among colleagues (past 5 years), and differences between women and men controlling for age and grade (unweighted and weighted analyses)** *

	Target of sexual harassment, sexual assault, and rape among colleagues						
	Women		Men		Difference between genders		
	Yes (%)†	Frequency‡	Yes (%)†	Frequency‡	Difference in frequency§¶	*P*	*d*
**Harassment: composite**	63.3	1.39(0.49)	23.7	1.14(0.47)	0.25 (0.20, 0.30)	< 0.001	0.50
Jokes with sexual content	52.7	1.95(1.01)	16.0	1.37(1.02)	0.58 (0.47, 0.69)	< 0.001	0.55
Displaying sexualized pictures	13.2	1.21(0.52)	3.8	1.07(0.53)	0.15 (0.09, 0.21)	< 0.001	0.26
E-comms, unwanted/sexual	16.3	1.25(0.55)	6.0	1.11(0.56)	0.14 (0.08, 0.21)	< 0.001	0.23
Physical advances, unwanted/sexual	29.2	1.42(0.62)	6.8	1.15(0.63)	0.27 (0.19, 0.35)	< 0.001	0.37
Unwanted/sexual talk	38.4	1.62(0.71)	9.6	1.20(0.69)	0.41 (0.32, 0.50)	< 0.001	0.48
Uninvited comments about body	40.3	1.63(0.65)	9.9	1.22(0.66)	0.41 (0.32, 0.50)	< 0.001	0.48
Ask for a date despite previous refusal	12.9	1.16(0.33)	2.9	1.06(0.34)	0.10 (0.05, 0.15)	< 0.001	0.21
Offered career opportunities for sex	5.0	1.07(0.25)	0.7	1.03(0.24)	0.05 (0.01, 0.09)	0.011	0.14
Threatened for refusing sexual favour	4.2	1.06(0.22)	0.4	1.02(0.21)	0.04 (0.01, 0.08)	0.022	0.13
Deliberately infringing body space	36.9	1.60(0.52)	7.5	1.18(0.51)	0.42 (0.33, 0.51)	< 0.001	0.48
**Assault: composite**	29.9	1.16(0.30)	6.9	1.06(0.32)	0.10 (0.06, 0.13)	< 0.001	0.31
Forced contact for career opportunities	10.9	1.16(0.48)	0.7	1.05(0.47)	0.12 (0.06, 0.17)	< 0.001	0.23
Touching, excluding genitals/breasts	27.6	1.39(0.70)	6.8	1.16(0.68)	0.22 (0.15, 0.30)	< 0.001	0.31
Touching of genitals/breasts	5.4	1.07(0.29)	0.6	1.02(0.29)	0.06 (0.02, 0.09)	< 0.001	0.18
Self-fondling by perpetrator	1.1	1.01(0.13)	0.1	1.00(0.13)	0.01 (−0.01, 0.02)	0.205	0.06
**Rape: composite**	0.8	1.01(0.11)	0.1	1.01(0.11)	0.00 (−0.01, 0.01)	0.767	0.02
Rape, workplace	0.4	1.00(0.08)	0.1	1.00(0.08)	0.00 (−0.01, 0.01)	0.949	0.00
Rape, other work contexts	0.8	1.01(0.16)	0.1	1.01(0.16)	0.00 (−0.01, 0.02)	0.616	0.03
**Weighted analyses**							
Harassment: composite	71.3	1.42(0.53)	29.0	1.21(0.53)	0.22 (0.13, 0.30)	< 0.001	0.36
Assault: composite	34.0	1.20(0.33)	10.3	1.09(0.32)	0.11 (0.06, 0.16)	< 0.001	0.29
Rape: composite	1.4	1.01(0.03)	0.0	1.00(0.02)	0.01 (0.00, 0.01)	0.020	0.17

Follow-up analyses testing the robustness of findings (for example with additional co-variates, non-parametric tests) yielded very similar results (also see †,¶). Additionally, as shown, weighted analyses yielded consistent results. †Proportion of women or men who witnessed this form of misconduct once or more in the past 5 years. These data provide an easily interpreted, descriptive illustration of the data. They are not integral to primary analyses, although follow-up analyses using this simplified (dichotomized, yes/no) form of the data yielded results that were highly consistent with those of primary analyses (for example, harassment composite, χ^2^(1) = 227.56, *P* < 0.001; assault composite, χ^2^(1) = 124.87, *P* < 0.001). ‡Mean(s.d.) frequency of being a target of each form of sexual misconduct (range of possible values 1–5). §Gender difference in mean frequency (95% c.i.), shown with *P* value and effect size (*d*) (general guide: *d* = 0.20, small effect; *d* = 0.50, medium effect)^18^. Overall, women and men differed in how often they experienced being a target of sexual misconduct (across harassment, assault and rape composites), multivariate F(3, 1428) = 31.83, *P* < 0.001, *d* = 0.52 (weighted: F(3, 733) = 8.89, *P* < 0.001, *d* = 0.38). Univariate tests indicated that women experienced sexual harassment, and sexual assault, more often than men (harassment, F(1, 1430) = 87.97, *P* < 0.001, *d* = 0.50; assault, F(1, 1430) = 33.13, *P* < 0.001, *d* = 0.31; weighted: harassment, F(1, 735) = 23.46, *P* < 0.001, *d* = 0.36; assault, F(1, 735) = 15.94, *P* < 0.001, *d* = 0.29) (*supplementary material*). ¶Based on means estimated at the mean of co-variates (age, grade). Where significant (*supplementary material*), older ages and grades predicted lower frequencies. Analyses included only respondents on doctor grades. This preserved the linear interpretability of this co-variate without drawing assumed equivalences across doctor, dental, and nursing (or other) grades. Follow-up analyses without co-variates and thus inclusion of respondents across all grades (doctor, dental, nursing, or other) yielded very similar results.

#### Unweighted analyses

Overall, women and men differed in how often they were a target of sexual misconduct. Univariate tests showed that women were targets of sexual harassment, and sexual assault, more often than men (*Table 3*).

#### Weighted analyses for NHS England

Results of weighted analyses provided further evidence that women experienced being a target of sexual harassment, and sexual assault, more often than men (*Table 3*), even when examined in a sample that mirrored the true representation of women and men in the NHS England surgical workforce.

### Adequacy of General Medical Council, NHS Trusts, and other organizations’ handling of sexual harassment and assault

*Table 4* shows respondents’ evaluations of whether the GMC, NHS Trusts, and other organizations are adequately addressing issues of sexual harassment and sexual assault. Women and men differed in their assessments, with women evaluating the adequacy of organizations’ handling of sexual misconduct consistently lower than men. Additional analyses (*supplementary material*) demonstrated that the perceived adequacy of these organizations also depended on the frequency of witnessing sexual misconduct, such that those who witnessed more sexual misconduct evaluated these organizations as less adequate (for example GMC evaluations, weighted analysis: *B* = −3.47 (95% c.i.: −4.43 to −2.51); *P* < 0.001, *η*_p_^2^ = 0.10) (*Table S3*).

**Table 4:** **Evaluations of whether the General Medical Council and other organizations are adequately handling issues of sexual misconduct: differences between women and men controlling for age, and grade (unweighted and weighted analyses)** *

	Is organization adequately addressing issues of sexual harassment and assault in our profession?						
	Women		Men		Difference between genders		
	Yes (%)†	Evaluation of adequacy‡	Yes (%)†	Evaluation of adequacy‡	Difference in evaluation§¶	*P*	*d*
British Medical Association	20.4	3.18(1.86)	57.8	4.47(1.87)	−1.29 (−1.55, −1.03)	< 0.001	0.66
General Medical Council	15.1	2.80(1.90)	48.6	4.07(1.91)	−1.28 (−1.54, −1.02)	< 0.001	0.64
Health Education England	22.8	3.18(1.91)	56.1	4.39(1.93)	−1.21 (−1.48, −0.93)	< 0.001	0.60
NHS Trusts	15.8	2.89(1.83)	44.9	4.12(1.84)	−1.23 (−1.47, −0.98)	< 0.001	0.64
Royal Colleges	31.1	3.55(1.95)	60.2	4.53(1.95)	−0.98 (−1.24, −0.73)	< 0.001	0.48
**Weighted analyses**							
British Medical Association	24.5	3.39(1.91)	59.2	4.29(1.86)	−0.90 (−1.30, −0.50)	< 0.001	0.42
General Medical Council	14.5	2.99(1.92)	38.6	3.65(1.85)	−0.66 (−1.06, −0.27)	< 0.001	0.30
Health Education England	18.7	3.18(1.95)	54.5	4.16(1.91)	−0.98 (−1.39, −0.57)	< 0.001	0.44
NHS Trusts	16.2	3.04(1.87)	43.9	4.06(1.83)	−1.02 (−1.39, −0.66)	< 0.001	0.48
Royal Colleges	30.6	3.51(1.92)	58.8	4.37(1.87)	−0.86 (−1.25, −0.48)	< 0.001	0.39

Follow-up analyses testing the robustness of findings (for example with additional covariates, multiple imputation) yielded very similar results. Additionally, as shown, weighted analyses yielded consistent results. †Total proportions of women and men who provided any generally positive evaluation of that organization (any value above the scale’s midpoint (4)). They provide an easily interpreted, descriptive illustration of the data. They are not integral to primary analyses, although follow-up analyses using this simplified (dichotomized, positive evaluation—yes/no) form of the data yielded results that were highly consistent with those of primary analyses (for example, for each organization, *χ^2^*(1) ≥ 81.92, *P* < 0.001). ‡Mean(s.d.) evaluations of the adequacy of the General Medical Council and other organizations in addressing issues of sexual misconduct (range of possible values 1–7). §Gender difference in mean evaluations (95% c.i.), shown with corresponding *P* values, and effect sizes (general guide: *d* = 0.20, small effect; *d* = 0.50, medium effect)^18^. ¶Based on means estimated at the mean of co-variates (age, grade). Where significant (*supplementary material*), older ages [higher grades] predicted more positive [negative] evaluations of the organization. Analyses included only respondents on doctor grades, to preserve the linear interpretability of this co-variate without drawing assumed equivalences across doctor, dental, and nursing (or other) grades. Follow-up analyses without co-variates and thus inclusion of respondents across all grades (doctor, dental, nursing, or other) yielded very similar results.

## Discussion

The results of this study on sexual misconduct over the past 5 years among the UK surgical workforce indicate that both sexual harassment and sexual assault may be commonplace in the UK surgical environment, and that rape happens. These are illegal and criminal acts. This study also provides robust evidence that women and men in the surgical workforce are living different realities. When around colleagues, women are both witnessing and being targets of sexual misconduct at higher rates than men. Moreover, results indicate that there is a widespread lack of confidence in the adequacy of key regulatory bodies and accountable organizations in handling these issues. Altogether, this reflects a serious issue within the NHS and broader surgical workforce, with implications for patient safety.

Strengths of this study include its large and diverse sample, with broad engagement from the surgical workforce, partly reflecting strategic efforts to recruit both individuals who have and those who have not experienced sexual misconduct. At the same time, the sensitivity of the survey content raised ethical and legal risks, and thus participant anonymity needed assurance. Therefore, to comply with ethically approved protocols, participants were asked to complete the survey in a single sitting. This may have resulted in some incomplete responses. Recruitment through listservs held by supporting organizations enabled a large and diverse sample, albeit individuals’ presence on multiple lists made subsequent response rate determination impossible.

Women remain a minority in surgery, comprising 28.3 per cent across all grades^15^, and 51.5 per cent of respondents in this survey. There is a skew to the responses; specifically, 63.1 per cent of responses were from consultants, who represent only 38.5 per cent of those in NHS England surgical grades. Specialty trainees comprised 20.2 per cent of the present participants and comprise 23.8 per cent of the surgical workforce in NHS England^15^. Participant numbers from specialty and specialist grade doctors and medical grades junior to specialty trainees, including medical students, were very low. These differences are consistent with work from the Royal Australasian College of Surgeons (RACS) in 2015^11^. They attest to the vulnerability and lack of psychological safety for those who are the most common targets of sexual misconduct, even if a survey is anonymous, whereas those who have reached consultant grade may feel more able to speak up^11^.

Most women (89.5 per cent) in this survey had witnessed sexual harassment, and 63.3 per cent had been a target of sexual harassment. Another 29.9 per cent of women had been the target of sexual assault (in the past 5 years). The RACS study^11^, and a large study of US surgical residents^19^, showed that 12 per cent of Australasian trainees (timescale undefined) and 10.3 per cent of US trainees (since beginning surgical residency) had experienced sexual harassment. Both studies^11,19^ included harassment from patients, but senior colleagues were the commonest perpetrators. A small 2021 UK survey^8^ reported that 48.8 per cent of UK female surgical trainees experienced sexual harassment by colleagues (during their training). These studies did not analyse sexual misconduct as separate items, simply reporting ‘sexual harassment’.

Men are also affected by sexual misconduct^20^. The results here, however, indicate that they are significantly less likely to witness it, or to be the target. For example, men’s experiences with sexual assault (17.1 per cent witnessed, 6.9 per cent targeted) is far lower than for women (35.9 per cent witnessed, 29.9 per cent targeted). Recent UK studies^21,22^ in healthcare have shown that sexual misconduct is predominantly carried out by men and towards women.

Both women and men in the present survey indicated exposure to ‘banter cultures’, yet research has revealed that these jokes are not innocuous, instead offering an important and subtle means for perpetrators to test the boundaries of their activities, identify like-minded individuals, and desensitize others^23^. These results also echo evidence that men tend to be less aware of such talk and its negative impact^24^. These differences, allied with gender disparities at senior levels in surgery, may be a significant contributory factor to the current failure to recognize these issues and bring about transformation.

A major concern arising from these results is the amount of sexual coercion reported by individuals, with women experiencing forced physical contact linked to career opportunities (16.6 per cent witnessed, 10.9 per cent targeted). Research identifies silence and silencing to be central to cultures that facilitate sexual violence^25^. These experiences divert attention from the needs of patients, and result in fear and intimidation, having further adverse consequences by distracting and disengaging learners and reducing performance in safety-critical contexts^2^.

Alongside instances of rape that occurred at work, participants in this survey reported rape by colleagues in other work-related contexts, including teaching spaces, conferences, and after-work events with colleagues. There are relatively few measures in place to protect the potentially vulnerable in settings such as conferences. These are also environments in which people may be away from home, alcohol is often available, and boundaries may be more easily transgressed by a perpetrator.

In the surgical profession, hierarchy mirrors power and responsibility. Arguably, an implicit aspect of becoming part of surgical culture is to not draw attention to sexual misconduct^20,26^. The surgical workplace is particularly vulnerable to sexual misconduct with its predominantly male senior workforce, use of strongly hierarchical structures, and high-stress environments^11^. Over time, sexualization of the workplace, through unwanted language, breaches of personal space, and physical violation, shifts accepted norms^27^. This normalization of unacceptable behaviour such as sexual misconduct leads current or would-be perpetrators to perceive tacit support for their sexualized behaviour. Without correction, cognitive reframing occurs, and in contexts of high stress and low emotional support, such as the surgical environment, this can undermine a doctor’s means of self-regulation. This allows inappropriate sexualized behaviours and misconduct to flourish^28,29^, where perpetrators target not only colleagues but are more likely to transgress sexual boundaries with patients^22,28^. The result is an unsafe working environment and an unsafe space for patients^2^.

Exposure to sexual misconduct deeply affects an individual’s psychological responses, altering their assumptions, beliefs, and expectations about themselves and others^20,25,30,31^. Sexual misconduct violates a target’s dignity and normalizes a hostile and abusive environment, affecting physical and mental well-being^2,19^. Those subjected to sexual misconduct are more likely to suffer burnout, which increases the risk of harm to patients^19^. Experiences of these illegal and criminal acts manifest in withdrawal from work both psychologically and physically as mental and physical health deteriorate. Even more harmful individual consequences can result, including depression, self-harm, and suicidal ideation^32^. Job satisfaction suffers, and so too does staff retention. These are serious events that potentially deter and derail women from realizing their career goals^33^.

Previous studies have shown women’s career decisions to be complex and multifactorial, recognizing the challenges to recruitment and retention posed by masculine environments, dysfunctional cultures, and inflexibility in surgery and surgical training^34^. The proportion of female consultant surgeons in the UK has increased by only 2 per cent since 2015, to 15 per cent. Numbers have risen in the specialty trainee grade, with women still comprising only 31 per cent of current trainees^15^, despite women outnumbering men at UK medical schools since 1996^35^. There are now more women in higher surgical training than at any point previously, yet the consultant body remains largely male. These survey results and the current gender imbalance indicate enduring significant risks of experiencing sexual misconduct.

Excellence in healthcare depends on teams, critically those in which all members feel safe and have a voice^36^. High-quality surgeon performance requires a safe learning environment, and dysfunctional teams are known to have poorer patient outcomes^37^. The failure to achieve diversity in the workforce, and the perpetuation of environments and atmospheres that normalize sexual misconduct in the workplace, pose a significant threat to staff and patient safety. Over time, perpetrators can become bolder, increasing the risks they pose to staff members and patients in their care^38,39^. Concerns have already been raised about the efficacy of external, fear-based, legal perpetrator controls in this context^21,22^. The present findings are likely to shake the confidence of the public in the surgical profession, in whom considerable trust is placed.

The UK workforce has not previously been asked about the perceived adequacy of accountable organizations in dealing with sexual misconduct. Concerns about sexism and racism have, however, been raised regarding the attitudes of the GMC, the BMA, and the Royal College of Surgeons of England^40–42^. In the present study, the percentage of women evaluating individual organizations as adequate ranged from 14.5 per cent (GMC) to 30.6 per cent (Royal Colleges). Individuals who more often witnessed sexual misconduct gave these key organizations even lower evaluations of adequacy, including women and men. This suggests that men who do recognize sexual misconduct also regard it as being handled inadequately. Participants’ low opinion is a concern, as research has demonstrated that perceived failure to hear concerns contributes to the decline in reporting, and the institutionalization of sexual violence^25^.

These findings require action. It is vital that the regulators, Colleges, employers, and training authorities come together to improve workforce and organizational cultures, and create adequate mechanisms to deal with perpetrators. This is a deeply serious issue that affects not just surgeons, and not just the UK healthcare workforce^1,2,7,11,26,28,32–34,37,38,43^. Cultural change in healthcare and accountable organizations is long overdue. Active bystander training, along with understanding and addressing barriers to reporting, are important next steps. Efforts to develop good practices that can reduce the inevitable harm from sexual misconduct are being developed in multiple working environments worldwide, and healthcare needs to learn from such efforts^11,19,20,26^.

The WPSMS proposes adopting a framework of zero tolerance for sexual misconduct in healthcare and robust mechanisms for dealing with perpetrators, modelled on recent work by the WHO^43^. Furthermore, in the UK, sexual safety policies should be implemented in healthcare, including NHS Trusts, with contravention treated as a serious incident and reported to the Care Quality Commission. The authors, on behalf of participants, the NHS workforce and patients, urge that immediate action be taken to make the surgical working environment, and healthcare in general, a safer place in which to work and be treated.

This study provides clear evidence of the differing realities that women and men experience as witnesses and targets of sexual misconduct by colleagues. While the exact prevalence of sexual misconduct in the surgical workforce cannot be determined from these data, this study provides a unique combination of statistically supported insights. These include how often individuals are targets of sexual misconduct among colleagues, how often it is witnessed, and how individuals evaluate the GMC and other organizations' handling of these issues. Robust evidence illustrating similar results across weighted and unweighted analyses strengthens validity of the demonstrated difference in women's and men's realities with sexual misconduct among colleagues.

Future studies, with greater support and long-term investment from surgical and healthcare organizations, should augment this foundational evidence by collecting representative samples of workforce members to better estimate prevalence of sexual misconduct. It will also be vital to track these issues over time, survey specific groups linked to medical schools and deaneries, and examine collaboratively issues of sexual misconduct via NHS staff surveys and GMC 360 revalidation questions.

## Supplementary Material

znad242_Supplementary_Data

## Data Availability

Data underlying the findings in this article are available at the Center for Open Science (https://osf.io/q4th5/). To preserve participant anonymity, this publicly accessible version of the data does not contain any free-text responses from participants, and some information has been aggregated or redacted to avoid potential identification of individual participants.
