# Corrigendum to: Sexual harassment, sexual assault and rape by colleagues in the surgical workforce, and how women and men are living different realities: observational study using NHS population-derived weights

**DOI:** 10.1093/bjs/znad385

**Published:** 2023-11-07

This is a corrigendum to: Christopher T Begeny, Homa Arshad, Tamzin Cuming, Daljit K Dhariwal, Rebecca A Fisher, Marieta D Franklin, Philippa C Jackson, Greta M McLachlan, Rosalind H Searle, Carrie Newlands, Sexual harassment, sexual assault and rape by colleagues in the surgical workforce, and how women and men are living different realities: observational study using NHS population-derived weights, *British Journal of Surgery*, Volume 110, Issue 11, November 2023, Pages 1518–1526, https://doi.org/10.1093/bjs/znad242

In the originally published version of this manuscript, there was a typographical error in the name of author Philippa C Jackson.

This has been corrected.

