# Supplementary material for: Sexual harassment, sexual assault and rape by colleagues in the surgical workforce, and how women and men are living different realities: observational study using NHS population-derived weights
Source: Br J Surg. 2023 Sep 12;110(11):1518–26. doi: 10.1093/bjs/znad242 (PMC10564399; doi:10.1093/bjs/znad242)
Supplement: znad242_Supplementary_Data [file znad242_supplementary_data.pdf]

Sexual Harassment, Sexual Assault and Rape by Colleagues in the Surgical Workforce – Women and Men are Living Different Realities: An Observational Study using NHS Population-derived Weights

Christopher T. Begeny, PhD [1]
Homa Arshad MB BChir, MA, FRCS [2]
Tamzin Cuming MBBS MA(Cantab), FRCS(Eng), FRCS(Gen Surg), MEd [3]
Daljit K Dhariwal BDS, FDSRCS(Eng), MBBCh, FRCS(Eng), FRCS(OMFS) [4]
Rebecca A Fisher BSc (Hons), MBChB, MEd, MFSTEd, MRCS [5]
Marieta D Franklin MBChB, MRes, FRCS(Orth) [6]
Philippa M Jackson MBBS, BSc, FRCS(Plast), MSc FHEA [7]
Greta M McLachlan MBChB, BSc, MRCS [8]
Rosalind H Searle PhD, MBA, C. Psychol., FBPS, FIAAP, FRSA, (Academic FCIPD) [9]
Carrie Newlands BDS, MBBCh, BAO, FDSRCS(Eng), FRCS(Ed), FRCS(OMFS), RCPath ME [10]

[1] Faculty of Health and Life Sciences, Department of Psychology, University of Exeter EX4 4QG UK
[2] Barts Bone and Joint Health, Barts Health National Health Service (NHS) Trust, The Royal London Hospital, Whitechapel, London E1 1BB UK
[3] Homerton University Hospital, Homerton Row, London E9 6SR UK
[4] Oxford University Hospitals NHS Foundation Trust, Nuffield Department of Surgical Sciences, University of Oxford. John Radcliffe Hospital, Headley Way, Headington, OX3 9DU UK
[5] School of Medical Sciences, Division of Medical Education, University of Manchester, M13 9ND UK
[6] Liverpool University Hospitals NHS Foundation Trust, L7 8XP UK
[7] North Bristol NHS Trust, BS10 5NB UK
[8] Frimley Health Foundation Trust, GU16 7UJ UK
[9] Adam Smith Business School, University of Glasgow, GU16 7UJ UK
[10] School of Biosciences and Medicine, University of Surrey, Stag Hill, Guildford, Surrey GU2 7XX, UK

Corresponding author
Christopher T. Begeny
University of Exeter
Washington Singer Laboratories
Perry Road, Exeter EX4 4QG
United Kingdom

ORCID ID
https://orcid.org/0000-0003-4734-8840

email
C.Begeny@exeter.ac.uk

Supplementary Materials - Index

Supplementary Methods

Measures *page 3*

Sexual harassment, assault, and rape among colleagues - Overview *page 3*

Definitions and guidance for responding *page 3*

Witnessing sexual harassment, assault, and rape among colleagues *page 5*

Table S1. Items assessing frequency of witnessing sexual harassment, sexual assault and rape among colleagues *page 6*

Being a target of sexual harassment, assault, and rape among colleagues *page 6*

Table S2. Items assessing frequency of being a target of sexual harassment, sexual assault and rape among colleagues *page 7*

Adequacy of GMC, NHS Trusts, and other organisations' handling of sexual harassment and assault *page 8*

Statistical Information *page 8*

Weighted analyses, mirroring representation of women and men in NHS-E surgical workforce *page 8*

Supplementary Results

Participant Information *page 9*

Gender Differences *page 9*

Witnessing sexual harassment, assault and rape among colleagues *page 9*

Being a target of sexual harassment, assault and rape among colleagues *page 10*

Adequacy of GMC, NHS Trusts, and other organisations' handling of sexual harassment and assault *page 11*

Additional Analyses *page 11*

Evaluations of GMC and other organisations' handling of sexual harassment and assault as a function of one's experiences with sexual misconduct *page 11*

Gender differences (no covariates) *page 11*

Table S3. Evaluations of organisations' handling of sexual harassment and assault as a function of one's experiences with sexual misconduct *page 13*

Supplementary Appendix

Participant information sheet and consent *page 15*

Debriefing form *page 17*

Supplementary Tables

Tables S1-S3 *Page 19*

Table S4 *page 19*

References *page 20*

Supplementary Methods

Measures

Sexual harassment, assault, and rape among colleagues - Overview

Participants were presented with a series of items used to assess their experiences witnessing, and being a target of, sexual harassment, sexual assault, and rape among work colleagues (for details, see proceeding subsections).

Definitions and guidance for responding

Prior to viewing these items, participants were given structured and uniform guidelines for responding to them. This included: (a) definitions of sexual harassment, sexual assault, and rape, (b) relevant (work-related) contexts and colleagues to consider when responding, and (c) when responding to witness-oriented questions, directions that stated:

In a separate question, we will ask if you have ever been the target or victim of these events. For this first question, please only indicate those events that you have witnessed, overheard or been present for (but not the direct target or victim of). Please do not include events that you have "heard about" but did not yourself directly witness, overhear, or be present for.

Below is the verbatim wording of the guidance and other relevant text that preceded these items:

> It is Important to Hear from Everyone
>
> For this survey, it is important to hear from as many members of the healthcare profession as possible. **Even if some questions do not seem relevant to you, we would really appreciate your responses**. This is important for getting a clearer picture of what's happening in our profession.
>
> …
> When responding to questions, it may be useful to consider the following
>
> **Definitions**
>
> **Sexual Harassment**
> Sexual harassment is unwanted behaviour of a sexual nature. The law (Equality Act 2010) protects the following people against sexual harassment at work:
>
> - Employees and workers
> - Contractors and self-employed people hired to personally do the work
> - Job applicants
>
> To be sexual harassment, the unwanted behaviour must have either:
>
> - Violated someone's dignity, whether it was intended or not
> - Created an intimidating, hostile, degrading, humiliating or offensive environment for them, whether it was intended or not [reference]
>
> If you have been harassed at work, sexual harassment can include:
>
> - Sexual comments or jokes
> - Physical behaviour, including unwelcome sexual advances or unwanted touching
> - Displaying pictures, photos or drawings of a sexual nature
> - Sending emails, messages, or other electronic communication with sexual content

You do not need to have previously refused or objected to someone's behaviour for it to be considered unwanted. [reference]

Sexual Assault & Rape
The definition of sexual violence, which encompasses sexual assault and rape, varies across the United Kingdom. The statutory definition of **sexual assault** under the Sexual Offences Act 2003 includes "**indecent exposure, sexual threats and unwanted touching** („less serious‟) and **rape or assault by penetration including attempts** („serious‟), by any person including a partner or family member." In Northern Ireland the definition of Sexual Violence used throughout the tackling Sexual Violence and Abuse strategy is "any behaviour perceived to be of a sexual nature which is unwanted and takes place without consent or understanding." In Scotland, the definition of violence against women as set out in the Scottish Government's 'Safer Lives: Changed Lives: A Shared Approach to Tackling Violence against Women in Scotland' – "Violence against women encompasses but is not limited to: Physical, sexual and psychological violence occurring in the family, within the general community or in institutions, including: domestic abuse, rape, incest and child sexual abuse." [reference, reference]

Assault by penetration
(1) A person (A) commits an offense if:
(a) he* intentionally penetrates the vagina or anus of another person (B) with a part of his body or anything else
(b) the penetration is sexual
(c) B does not consent to the penetration, and
(d) A does not reasonably believe that B consents.
(2) Whether a belief is reasonable is to be determined having regard to all the circumstances, including any steps A has taken to ascertain whether B consents.

Sexual assault
(1) A person (A) commits an offense if:
(a) he* intentionally touches another person (B)
(b) the touching is sexual
(c) B does not consent to the touching, and
(d) A does not reasonably believe that B consents.
(2) Whether a belief is reasonable is to be determined having regard to all the circumstances, including any steps A has taken to ascertain whether B consents.

Rape
(1) A person (A) commits an offense if:
(a) he* intentionally penetrates the vagina, anus or mouth of another person (B) with his penis
(b) B does not consent to the penetration, and
(c) A does not reasonably believe that B consents.
(2) Whether a belief is reasonable is to be determined having regard to all the circumstances, including any steps A has taken to ascertain whether B consents.
[reference]

*Some definitions which are in current legal usage do not reflect an understanding that gender, and gender pronouns, are not binary. They also do not always reflect an understanding that an individual's gender is not synonymous with, nor defined by, any particular primary or secondary sex characteristics. For the purposes of definitions in this survey, rape can be committed by any person with a penis, and sexual assault can be committed by any person.

Victim, Survivor
A victim is someone who has suffered as a result of someone else's actions or beliefs, or as a result of unpleasant circumstances. [reference]

The UK Government Code of Practice for victims of crime "acknowledges that the terms

‘complainant’ and ‘survivor’ are often used in the criminal justice system to describe a person who has made a criminal allegation to the police. However, for the purpose of this Code, the definition of a ‘victim’ is: a person who has suffered harm, including physical, mental or emotional harm or economic loss which was directly caused by a criminal offence.” [reference]

A survivor (of sexual misconduct) is someone who has gone or is going through the recovery process. [reference] In this survey, we do not intend to impose these terms in a prescriptive manner. We acknowledge and respect that individuals will differ in their use of these terms.

Witness
In general, a witness is someone who sees an event happening. [reference] In this survey, we do not to use this term in a legal sense. It more broadly refers to someone who has seen, overheard or been present for an event. *It excludes hearsay.*

When responding to the questions below, please consider the following

Work-related Contexts & Colleagues

Work-related Contexts
For the questions below, please consider your experiences in work-related contexts. This includes:

- The workplace (e.g., at the hospital)
- Teaching-related spaces
- Conferences
- After-work social events (with work colleagues)
- Online / Social networking (with work colleagues)

If you have had any relevant experiences that occurred in other settings (e.g., home settings, parties) but still among work colleagues, please explain in the open-text boxes.

Work Colleagues
Work colleagues include:

- Any health professional
- Any other colleague or employee of a health-related organisation
- Students (e.g., medical, dental, nursing)

Witnessing sexual harassment, assault, and rape among colleagues

Participants were presented with 16 items used to assess their experiences witnessing sexual harassment, sexual assault, and rape among work colleagues. Table S1 shows their verbatim wording. Items were adapted from previous work [1,2] and incorporated feedback from healthcare professionals who have themselves witnessed and been targets of sexual misconduct, in part to ensure items were relevant and fitting to the professional context.

The items were preceded with the question/stem, “In the past 5 years (in any work-related context), how often have you witnessed, overheard, or been present for...” (consistent with past work using a multi-year timeframe).[3] Responses were measured on a 1 - 5 scale: *Never*, *Rarely (less than once a year)*, *Sometimes (a few times a year)*, *Often (a few times a month), Very Often (a few times a week, or more*).

Table S1. Items assessing frequency of witnessing sexual harassment, sexual assault and rape among colleagues [a]

Form of Sexual Misconduct	*Response Item*
Harassment	(Witness)
1	Jokes with sexual content
2	Displaying of pictures of a sexual / inappropriate nature in public or shared spaces (e.g., washrooms/changing areas, departmental offices, during a presentation)
3	Emails, text messages, or other electronic communications with sexual content that was unwanted
4	Unwanted/inappropriate physical advances of a sexual kind
5	Unwanted sexual talk or comments (e.g., unwanted or inappropriate sexual remarks about an individual, or about an individual's involvement in sexual activities)
6	Uninvited/inappropriate comments about someone's anatomy/body parts (including both seemingly 'positive' and negative comments)
7	Someone being asked for a date on one or more occasions after having already refused that person
8	Offer of promotion or other work-related advantage or advancement in exchange for a sexual favour (e.g., offering a reward or special treatment if someone cooperated sexually)
9	Threats of punishment or adverse consequences for refusal of a sexual favour
10	Deliberate infringing on someone's body space
Assault	(Witness)
1	Being forced to partake in physical contact in exchange for training development / career development (e.g., an unwanted or uninvited hug, kiss on the cheek, arm around the shoulder, or a more extreme or overtly sexual type of behaviour)
2	Touching of someone's body without consent, including any area of the body (except genitals and/or breast tissue)
3	Touching of someone's genitals and/or breast tissue without consent
4	Self-fondling by perpetrator (e.g., masturbation)
Rape	(Witness)
1	Rape in the workplace
2	Rape in any other work-related context (e.g., teaching spaces, conferences, after-work social events with colleagues)

[a] Participants were initially asked if they had ever witnessed these instances/items while working or training in the healthcare profession (*No / Yes*) and to enhance the flow and efficiency of the survey, if a participant responded *No*, those items were not presented when asking about frequency over the past 5 years. Instead, the corresponding 'past 5 years' item was automatically coded as 1 [*Never*] for any pertinent analyses; Inter-item reliabilities (unweighted): harassment/assault, α = .88/.66; rape, r = .76.

Being a target of sexual harassment, assault, and rape among colleagues

We also assessed participants' experiences being a target of sexual harassment, sexual assault, and rape. We used the same 16 items, response scale, and procedural steps used to assess witnessing such instances. Items were adjusted appropriately to ask about being a target (vs. witness; e.g., *Uninvited/inappropriate comments about* your [vs. someone's] *anatomy/body parts…*), and the question/stem asked, "In the past 5 years (in any work-related context), how often have you been the target or victim/survivor of..." Table S2 shows the verbatim wording of items.

Table S2. Items assessing frequency of being a target of sexual harassment, sexual assault and rape among colleagues [a]

**Form of Sexual Misconduct**	
Response Item	
Harassment	(Target)
1	Jokes with sexual content
2	Displaying of pictures of a sexual / inappropriate nature in public or shared spaces (e.g., washrooms/changing areas, departmental offices, during a presentation)
3	Emails, text messages, or other electronic communications with sexual content that was unwanted
4	Unwanted/inappropriate physical advances of a sexual kind
5	Unwanted sexual talk or comments (e.g., unwanted or inappropriate sexual remarks about you, or about your involvement in sexual activities)
6	Uninvited/inappropriate comments about your anatomy/body parts (including both seemingly 'positive' and negative comments)
7	Being asked for a date on one or more occasions after having already refused that person
8	Offer of promotion or other work-related advantage or advancement in exchange for a sexual favour (e.g., offering a reward or special treatment if you cooperated sexually)
9	Threats of punishment or adverse consequences for refusal of a sexual favour
10	Deliberate infringing on your body space
Assault	(Target)
1	Being forced to partake in physical contact in exchange for training development / career development (e.g., an unwanted or uninvited hug, kiss on the cheek, arm around the shoulder, or a more extreme or overtly sexual type of behaviour)
2	Touching of your body without consent, including any area of the body (except genitals and/or breast tissue)
3	Touching of your genitals and/or breast tissue without consent
4	Self-fondling by perpetrator (e.g., masturbation)
Rape	(Target)
1	Rape in the workplace
2	Rape in any other work-related context (e.g., teaching spaces, conferences, after-work social events with colleagues)

[a] Participants were initially asked if they had ever been the target of these instances/items while working or training in the healthcare profession (*No / Yes*) and to enhance the flow and efficiency of the survey, if a participant responded *No*, those items were not presented when asking about frequency over the past 5 years. Instead, the corresponding 'past 5 years' item was automatically coded as 1 [*Never*] for any pertinent analyses; Inter-item reliabilities (unweighted): harassment/assault, $\alpha = .90/.63$; rape, $r = .88$.

These 16 items (Tables S1-S2) were generally categorised in line with the same UK-based definitions of sexual harassment, assault and rape presented to participants (drawn partly from legal and legislative texts and other relevant advisory organisations)[4–9] and indeed participants viewed the items under these categorical subheadings, though as in previous research,[3] harassment items were not explicitly labelled as such (presented under that subheading) but simply presented ahead of the other items (though as previously described, a definition of harassment was provided).

It is important to recognize that even within the UK there are differing definitions for these concepts (e.g., in legal and legislative texts). Academic literature provides still other potential bases for distinguishing these experiences (e.g., unwanted sexual attention vs. sexual coercion)[1,2]. As such, it is difficult to provide universally-accepted conceptual boundaries around these forms of sexual misconduct – nor is this the central aim of the present study. In light of this, in primary unweighted analyses results of both individual items (maximally agnostic of an item's underlying construct) are included and composites that correspond to the categorisation of items as presented within the survey. It is also important to note that: (a) results across individual items and their corresponding composite scores were highly consistent, and (b) these data are publicly available to explore other questions that are of interest to particular readers (e.g., fit of the data to unidimensional- vs. correlated- vs. second-order- vs. bi-factor structure models).

Adequacy of GMC, NHS Trusts, and other organisations' handling of sexual harassment and assault

Participants were presented with a list of organisations and asked, "For the organisations and institutions listed below, do you believe they are adequately addressing issues of sexual harassment and assault in our profession?" Participants responded on a scale from 1 - *No, Absolutely Not* to 7 - *Yes, Absolutely* (additional response option: *N/A; Don't Know, or Not Applicable*).

The question was intentionally framed to assess whether organisations' handling of sexual harassment and assault was considered *adequate* – reflecting a minimal threshold for positive evaluation (i.e., responses above the midpoint). We asked participants to evaluate several organisations and other relevant working domains/referents: British Medical Association, General Medical Council, General Dental Council, NHS Trusts, Public Health England, Royal Colleges, Deaneries / Health Education England, Universities, Nursing & Midwifery Council, Your Current Place of Work, The Profession Overall (regulatory bodies, org. leaders, managers, colleagues, etc), Other (please specify): [open text box].

Statistical Information

Primary analyses involved (multivariate) analyses of covariance to test for mean gender differences (0 *women*, 1 *men*). Additional analyses using linear (OLS) regression tested whether individuals' evaluations of the GMC and other organisations were further explained by the frequency at which they witnessed and/or were targets of sexual misconduct (predictors mean centred). All analyses were conducted in SPSS v28 using the GLM subcommand with covariates: participant age, grade (complete-case analysis). All tests were two tailed, with a significance level of 0.05. Follow-up analyses assessed the robustness of findings, including through the use of additional covariates, bootstrapping, and multiple imputation. These and other follow-up analyses yielded highly consistent results with those evinced in primary analyses.

Weighted analyses, mirroring representation of women and men in NHS-E surgical workforce

To help mitigate biased estimates and produce a sample for analyses that mirrored the surgical population by gender, grade, and key subspecialties, population data on the NHS surgical workforce in England (NHS-E) [10] was used to generate case weights for a corresponding set of participants who were part of the NHS-E surgical workforce. Most notably, this meant that while the proportions of women and men were approximately equal in the unweighted analyses, in weighted analyses the proportions reflected those in the population: 27.7% women and 72.3% men.

Weights were calculated using iterative proportional fitting (rake weighting; using the SPSSINC_RAKE extension) and the marginal distributions for: (1) gender: 27.7% women, 72.3% men; (2) grade: 38.9% consultants, 9.4% associate

specialist/specialty doctor/staff grade, 24.1% specialty registrar, and 27.7% core training;[†] and (3) key subspecialties: 31.5% in general surgery, 27.5% in trauma and orthopaedic surgery, 41.0% in other, smaller subspecialties.[‡] The raking procedure converged in 8 iterations and 98.8% of weights were less than W_{UL} = median(W) + (5 * IQR(W)) = 6.24; median(W) = 0.603, IQR(W) = 1.128. Weights were normalised to preserve equivalent sample sizes and degrees of freedom when running like-for-like analyses with and without weights (described in main text). Weights were implemented via the WLS [weighted least squares] subcommand within GLM. Primary analyses used these normalised weights as originally generated. Very similar results emerged in analyses using: (1) trimmed weights, wherein weights > W_{UL} (*n* = 9 participants [1.2% of sample]) were trimmed to W_{UL} (all other weights adjusted accordingly to preserve normalisation), and (2) weights based on joint rather than marginal distributions (post-stratification weights).

Supplementary Results

Participant Information

Of the 1,704 women and men who completed the study, 1,434 (51.5% women) provided responses that enabled inclusion in primary analyses (remainder of participants provided responses that enabled inclusion in at least some follow-up analyses; e.g., those akin to primary analyses but without covariates).[§] The two largest subspecialties in the surgical workforce [10] were also the two largest represented in the sample: trauma and orthopaedic surgery (31.8%) and general surgery (18.5%). Similarly, the two largest grades in the surgical workforce [doctor-level][10] were also the two largest represented in the sample: consultants (63.1%) and specialty trainees (20.2%; see Table 1 for additional information). For weighted analyses, participants from the corresponding NHS surgical population were eligible (*n* = 756).

Gender Differences

Witnessing sexual harassment, assault and rape among colleagues

Table 2 (main text) provides the overall proportions of women and men who witnessed sexual harassment, assault and rape in the past 5 years (once or more). It also provides the mean frequencies for women and men, in both unweighted and weighted samples, and corresponding tests of gender differences.

Unweighted Analyses

Overall, women and men differed in how often they witnessed sexual misconduct (across harassment, assault and rape composites), *multivariate* $F(3, 1410) = 22.67$, $p < .001$, $d = .44$ (covariates: age, $F(3, 1410) = 10.86$, $p < .001$, $d = .31$; grade $F(3, 1410) = 4.81$, $p = .002$, $d = .20$). As outlined in Table 2, univariate tests showed that women witnessed sexual harassment more often than men, $F(1, 1412) = 63.89$, $p < .001$, $d = .42$ (covariates: age, $F(1, 1412) = 21.10$, $p <$

[†] The current study did not aim to exclusively recruit NHS-based doctors and so survey response options for doctor grades were mapped onto those utilised in NHS-based population data as follows, with survey-based grades in italics: (a) Consultant [*Consultant (Doctor)*], (b) Associate Specialist/Specialty Doctor/Staff Grade/Hospital Practitioner/Clinical Assistant [*Trust Grade Registrar/SAS/Specialty Doctor*], (c) Specialty Registrar [*Specialty Trainee/Post-CCT Fellow*], (d) Core Training [*Core Trainee/Trust Grade SHO/Clinical Fellow*]. NHS data on 'Other and Local HCHS Doctor Grades' were not used in generating weights because this 'other' category could not be readily mapped onto nor sensibly ranked among the other grades. Very few participants were foundation doctors (Year 1/2) and so were collapsed into Core Training for the purpose of generating weights.

[‡] We utilised three key subspecialty categories for generating weights and all corresponding analyses. This included the two largest subspecialties within the surgical workforce, general surgery and T&O, which were also the two largest within our sample, and a third category of other smaller subspecialties collapsed. This approach ensured that weights provided a reasonable balance between precision/decreased bias and size of sampling variances.

[§] Additional rows of data (often largely blank) were not included in any analyses (601; e.g., rows reflecting individuals who did not answer any/nearly any questions, did not exclusively identify as a woman or man, did not specify a gender, and/or were under 18 years old). Missing data (sample for primary unweighted analyses) are as follows, with W=Witnessed, T=Target of, H=Sexual Harassment, A=Sexual Assault, R=Rape, C=Composite Measure, I=Individual Items, E=Evaluated Adequacy of organisations, BMA=British Medical Association, GMC=General Medical Council, HEE=Health Education England, NHS=NHS Trusts, RC=Royal Colleges: THC: 0, TAC: 0, TRC: 0, WHC: 2, WAC: 3, WRC: 16, THI: 2-20, TAI: 2-15, TRI: 0-18, WHI: 6-36, WAI: 6-18, WRI: 17-45, E-BMA: 174, E-GMC: 177, E-HEE: 186, E-NHS: 177, E-RC: 177; follow-up analyses using multiple imputation on E-items were consistent with corresponding results in primary analyses.

.001, $d = .25$; grade $F(1, 1412) = 0.30$, $p = .59$, $d = .03$), and sexual assault more often than men, $F(1, 1412) = 32.31$, $p < .001$, $d = .30$ (covariates: age, $F(1, 1412) = 0.65$, $p = .42$, $d = .05$; grade $F(1, 1412) = 4.43$, $p = .04$, $d = .11$). The frequency of witnessing rape was relatively low overall and did not differ on average for women and men, $F(1, 1412) = 0.56$, $p = .45$, $d = .04$ (covariates: age, $F(1, 1412) = 0.28$, $p = .60$, $d = .25$; grade $F(1, 1412) = 5.60$, $p = .02$, $d = .13$).

As shown in Table 2, tests of individual items further demonstrated that women witnessed various forms of sexual harassment more often than men (*multivariate* $F(10, 1372) = 14.98$, $p < .001$, $d = .66$; covariates: age, $F(10, 1372) = 7.57$, $p < .001$, $d = .47$; grade, $F(10, 1372) = 2.59$, $p = .004$, $d = .28$), and most instances of sexual harassment more often than men (*multivariate* $F(4, 1403) = 9.78$, $p < .001$, $d = .33$; covariates: age, $F(4, 1403) = 1.25$, $p = .29$, $d = .13$; grade, $F(4, 1403) = 1.13$, $p = .34$, $d = .11$; no mean differences on rape items, *multivariate* $F(2, 1383) = 1.82$, $p = .16$, $d = .11$; covariates: age, $F(2, 1383) = 0.29$, $p = .75$, $d = .04$; grade, $F(2, 1383) = 2.80$, $p = .06$, $d = .13$).

Weighted analyses (NHS England)

Results of weighted analyses were highly consistent with unweighted analyses. Overall, women and men differed in how often they witnessed sexual misconduct (across harassment, assault and rape composites), *multivariate* $F(3, 727) = 13.98$, $p < .001$, $d = .48$ (covariates: age, $F(3, 727) = 6.56$, $p < .001$, $d = .33$; grade $F(3, 727) = 14.21$, $p < .001$, $d = .48$). As shown in Table 2, women witnessed sexual harassment more often than men, $F(1, 729) = 26.37$, $p < .001$, $d = .38$ (covariates: age, $F(1, 729) = 2.86$, $p = .09$, $d = .13$; grade $F(1, 729) = 3.61$, $p = .06$, $d = .14$), and sexual assault more often than men, $F(1, 729) = 8.48$, $p = .004$, $d = .21$ (covariates: age, $F(1, 729) = 0.05$, $p = .82$, $d = .02$; grade $F(1, 729) = 15.58$, $p < .001$, $d = .29$). Frequencies of witnessing rape did not differ on average for women and men, $F(1, 729) = 0.68$, $p = .41$, $d = .06$ (covariates: age, $F(1, 729) = 6.64$, $p = .01$, $d = .19$; grade $F(1, 729) = 36.82$, $p < .001$, $d = .45$).

Being a target of sexual harassment, assault and rape among colleagues

Table 3 (main text) provides the overall proportions of women and men who witnessed sexual harassment, assault and rape in the past 5 years (once or more; for descriptive purposes). It also provides the mean frequencies for women and men, in both unweighted and weighted samples, and corresponding tests of gender differences.

Unweighted Analyses

Overall, women and men differed in how often they witnessed sexual misconduct (across harassment, assault and rape composites), *multivariate* $F(3, 1428) = 31.83$, $p < .001$, $d = .52$ (covariates: age, $F(3, 1428) = 3.81$, $p = .01$, $d = .18$; grade $F(3, 1428) = 2.17$, $p = .09$, $d = .14$). As outlined in Table 3, univariate tests showed that women experienced sexual harassment more often than men, $F(1, 1430) = 87.97$, $p < .001$, $d = .50$ (covariates: age, $F(1, 1430) = 8.62$, $p = .003$, $d = .16$; grade $F(1, 1430) = 5.53$, $p = .02$, $d = .13$), and sexual assault more often than men, $F(1, 1430) = 33.13$, $p < .001$, $d = .31$ (covariates: age, $F(1, 1430) = 8.26$, $p = .004$, $d = .16$; grade $F(1, 1430) = 4.98$, $p = .03$, $d = .11$). Frequencies of experiencing rape did not differ on average for women and men, $F(1, 1430) = 0.09$, $p = .77$, $d = .02$ (covariates: age, $F(1, 1430) = 0.01$, $p = .92$, $d = .00$; grade $F(1, 1430) = 2.22$, $p = .14$, $d = .09$).

As shown in Table 3, tests of individual items further illustrated that women experienced each instance of sexual harassment more often than men (*multivariate*, $F(10, 1389) = 12.97$, $p < .001$, $d = .61$; covariates: age, $F(10, 1389) = 2.14$, $p = .02$, $d = .25$; grade $F(10, 1389) = 2.76$, $p = .002$, $d = .29$), and most instances of sexual harassment more often than men (*multivariate* $F(4, 1401) = 9.15$, $p < .001$, $d = .32$; covariates: age, $F(4, 1401) = 4.09$, $p = .003$, $d = .22$; grade, $F(4, 1401) = 2.58$, $p = .04$, $d = .17$; no mean differences on rape items, *multivariate* $F(2, 1411) = 0.69$, $p = .50$, $d = .06$; covariates: age, $F(2, 1411) = 0.03$, $p = .97$, $d = .01$; grade, $F(2, 1411) = 1.13$, $p = .32$, $d = .09$).

Weighted analyses (NHS England)

Results of weighted analyses were again highly consistent with unweighted analyses. Overall, women and men differed in how often they experienced sexual misconduct (across harassment, assault and rape composites), *multivariate* $F(3, 733) = 8.89$, $p < .001$, $d = .38$ (covariates: age, $F(3, 733) = 0.47$, $p = .70$, $d = .09$; grade $F(3, 733) = 5.28$, $p = .001$, $d = .29$). As outlined in Table 3, univariate tests showed that women experienced sexual harassment more often than men, $F(1, 735) = 23.46$, $p < .001$, $d = .36$ (covariates: age, $F(1, 735) = 0.97$, $p = .32$, $d = .06$; grade $F(1, 735) = 15.77$, $p < .001$, $d = .29$), and sexual assault more often than men, $F(1, 735) = 15.94$, $p < .001$, $d = .29$ (covariates: age, $F(1, 735) = 0.19$, $p = .67$, $d = .03$; grade $F(1, 735) = 11.62$, $p < .001$, $d = .26$). Frequencies of experiencing rape also differed modestly between women and men, $F(1, 735) = 5.41$, $p = .02$, $d = .17$ (covariates: age, $F(1, 735) = 0.02$, $p = .90$, $d = .01$; grade $F(1, 735) = 0.30$, $p = .58$, $d = .04$).

Adequacy of GMC, NHS Trusts, and other organisations' handling of sexual harassment and assault

Unweighted analyses

Table 4 (main text) shows women's and men's evaluations of whether the GMC and other organisations are adequately addressing issues of sexual harassment and assault. Analyses of covariance showed that women and men differed in their evaluations: BMA, $F(1, 856) = 93.42$, $p < .001$, $d = .66$ (covariates: age $F(1, 856) = 26.84$, $p < .001$, $d = .35$; grade $F(1, 856) = 5.37$, $p = .02$, $d = .16$); GMC, $F(1, 916) = 93.10$, $p < .001$, $d = .64$ (covariates: age $F(1, 916) = 34.54$, $p < .001$, $d = .39$; grade $F(1, 916) = 2.66$, $p = .10$, $d = .11$); HEE, $F(1, 815) = 72.56$, $p < .001$, $d = .60$ (covariates: age $F(1, 815) = 24.92$, $p < .001$, $d = .35$; grade $F(1, 815) = 0.85$, $p = .36$, $d = .06$); NHS Trusts, $F(1, 977) = 99.66$, $p < .001$, $d = .64$ (covariates: age $F(1, 977) = 12.04$, $p < .001$, $d = .22$; grade $F(1, 977) = 0.01$, $p = .93$, $d = .02$); Royal Colleges, $F(1, 955) = 55.82$, $p < .001$, $d = .48$ (covariates: age $F(1, 955) = 22.78$ $p < .001$, $d = .31$; grade $F(1, 955) = 0.70$, $p = .40$, $d = .06$). As shown in Table 4, women's evaluations were consistently lower than men's. Moreover, descriptively, the percentage of women evaluating these organisations as generally 'adequate' (anywhere above the scale's midpoint) was, at most, 31.1% (Royal Colleges; men's evaluation: 60.2%). The GMC received the lowest evaluations of adequacy, at 15.1% (men's evaluation: 48.6%). Evaluations of NHS Trusts were similarly low, at 15.8% (men's evaluation: 44.9%).

Weighted analyses (NHS England)

Table 4 shows women's and men's evaluations of whether the GMC and other organisations are adequately addressing issues of sexual harassment and assault. Analyses of covariance showed that women and men differed in their evaluations: BMA, $F(1, 456) = 19.82$, $p < .001$, $d = .42$ (covariates: age $F(1, 456) = 8.93$, $p = .003$, $d = .28$; grade $F(1, 456) = 0.25$, $p = .62$, $d = .06$); GMC, $F(1, 483) = 10.97$, $p < .001$, $d = .30$ (covariates: age $F(1, 483) = 17.32$, $p < .001$, $d = .38$; grade $F(1, 483) = 0.28$, $p = .60$, $d = .06$); HEE, $F(1, 456) = 21.95$, $p < .001$, $d = .44$ (covariates: age $F(1, 456) = 14.42$, $p < .001$, $d = .36$; grade $F(1, 456) = 0.18$, $p = .67$, $d = .04$); NHS Trusts, $F(1, 526) = 30.27$, $p < .001$, $d = .48$ (covariates: age $F(1, 526) = 12.65$, $p < .001$, $d = .31$; grade $F(1, 526) = 0.98$, $p = .32$, $d = .09$); Royal Colleges, $F(1, 511) = 19.25$, $p < .001$, $d = .39$ (covariates: age $F(1, 511) = 6.51$ $p = .01$, $d = .23$; grade $F(1, 511) = 0.22$, $p = .64$, $d = .04$). As shown in Table 4, women's evaluations were consistently lower than mens. Moreover, descriptively, the percentage of women evaluating these organisations as generally 'adequate' (anywhere above the scale's midpoint) was, at most, 30.6% (Royal Colleges; men's evaluation: 58.8%). The GMC received the lowest evaluations of adequacy, at 14.5% (men's evaluation: 38.6%). Evaluations of NHS Trusts were similarly low, at 16.2% (men's evaluation: 43.9%).

Additional Analyses

Evaluations of GMC and other organisations' handling of sexual harassment and assault as a function of one's experiences with sexual misconduct

Following primary analyses, preliminarily tests were performed whether individuals' evaluations of the GMC and other organisations differed not only as a function of their gender but how often they experienced being a target of, or witnessing, sexual misconduct (with interaction terms). As shown in Table S3, in both weighted and unweighted analyses, gender remained a significant predictor. Women's evaluations of the adequacy of these organisations were lower than men's. Importantly, however, the frequency at which individuals witnessed sexual misconduct was also a consistent and particularly strong predictor of individuals' evaluations. When individuals witnessed sexual misconduct occurring among colleagues more often, their evaluations of these organisations' adequacy were consistently lower.

Gender differences (no covariates)

Paralleling primary unweighted analyses, follow-up tests of gender differences without covariates and thus inclusion of participants across all grades (not just doctor grades but also dental, nursing, or other) yielded very similar results.

Witnessing sexual harassment, assault and rape among colleagues

Overall, women and men differed in how often they witnessed sexual misconduct (across harassment, assault and rape composites), *multivariate* $F(3, 1643) = 46.89$, $p < .001$, $d = .59$. Univariate tests showed that women witnessed sexual harassment more often than men, $F(1, 1645) = 138.10$, $p < .001$, $d = .58$, and sexual assault more often than men, $F(1, 1645) = 60.99$, $p < .001$, $d = .39$. The frequency of witnessing rape was relatively low overall and did not differ on average for women and men, $F(1, 1645) = 3.74$, $p = .05$, $d = .09$.

Being a target of sexual harassment, assault and rape among colleagues

Overall, women and men differed in how often they witnessed sexual misconduct (across harassment, assault and rape composites), *multivariate* $F(3, 1649) = 68.29$, $p < .001$, $d = .71$. Univariate tests showed that women experienced sexual harassment more often than men, $F(1, 1651) = 194.84$, $p < .001$, $d = .69$, and sexual assault more often than men, $F(1, 1651) = 86.51$, $p < .001$, $d = .46$. Frequencies of experiencing rape did not differ on average for women and men, $F(1, 1651) = 1.51$, $p = .22$, $d = .06$.

Adequacy of GMC, NHS Trusts, and other organisations' handling of sexual harassment and assault

Women and men differed in their evaluations of whether the GMC and other organisations are adequately addressing issues of sexual harassment and assault, with women's evaluations consistently lower than men's: BMA, $F(1, 956) = 176.68$, $p < .001$, $d = .86$; GMC, $F(1, 1014) = 184.70$, $p < .001$, $d = .85$; HEE, $F(1, 916) = 147.59$, $p < .001$, $d = .80$; NHS Trusts, $F(1, 1121) = 177.91$, $p < .001$, $d = .80$; Royal Colleges, $F(1, 1073) = 128.30$, $p < .001$, $d = .69$.

Table S3. Evaluations of organisations' handling of sexual harassment and assault as a function of one's experiences with sexual misconduct

	**British Medical Association**			**General Medical Council**			**Health Education England**			**NHS Trusts**			**Royal Colleges**		
	B	95% CI	η_p^2	*B*	95% CI	η_p^2	*B*	95% CI	η_p^2	*B*	95% CI	η_p^2	*B*	95% CI	η_p^2
**Gender** (0 *woman*, 1 *man*)	1.08***	[0.81 to 1.36]	.066	1.11***	[0.83 to 1.39]	.062	0.95***	[0.65 to 1.24]	.047	0.88***	[0.62 to 1.14]	.044	0.70***	[0.43 to 0.98]	.026
**Witnessing** Sexual Misconduct [a]	-1.93***	[-2.39 to -1.47]	.075	-1.89***	[-2.36 to -1.42]	.064	-1.97***	[-2.44 to -1.49]	.076	-1.90***	[-2.32 to -1.48]	.075	-2.21***	[-2.66 to -1.75]	.087
**Being a Target** of Sexual Misconduct [a]	-0.09	[-0.82 to 0.64]	.000	-0.16	[-0.94 to 0.61]	.000	-0.47	[-1.23 to 0.30]	.002	-0.92**	[-1.65 to -0.19]	.006	-0.03	[-0.83 to 0.76]	.000
*Gender * Witness*	-0.84	[-1.75 to 0.06]	.004	-1.35**	[-2.28 to -0.42]	.009	0.77	[-0.82 to 2.35]	.001	-1.20**	[-2.04 to -0.37]	.008	-0.87	[-1.78 to 0.03]	.004
*Gender * Target*	1.19	[-0.28 to 2.66]	.003	1.22	[-0.34 to 2.77]	.003	-1.26**	[-2.21 to -0.32]	.009	1.15	[-0.35 to 2.65]	.002	1.11	[-0.51 to 2.73]	.002
*Witness * Target*	0.41**	[0.10 to 0.72]	.008	0.53**	[0.20 to 0.87]	.011	0.61***	[0.29 to 0.93]	.017	0.86**	[0.32 to 1.41]	.010	0.48**	[0.15 to 0.82]	.008
*Gender * Witness * Target*	-0.43	[-1.06 to 0.21]	.002	-0.24	[-0.92 to 0.43]	.001	-0.11	[-0.79 to 0.57]	.000	0.03	[-1.12 to 1.18]	.000	-0.20	[-0.89 to 0.49]	.000
Age	0.12***	[0.06 to 0.19]	.014	0.15***	[0.08 to 0.22]	.019	0.14***	[0.06 to 0.22]	.016	0.04	[-0.03 to 0.10]	.001	0.09**	[0.03 to 0.16]	.008
Grade	-0.11*	[-0.21 to -0.02]	.006	-0.07	[-0.17 to 0.03]	.002	-0.06	[-0.16 to 0.05]	.001	0.01	[-0.08 to 0.10]	.000	-0.03	[-0.12 to 0.07]	.000

Weighted Analyses

	**British Medical Association**			**General Medical Council**			**Health Education England**			**NHS Trusts**			**Royal Colleges**		
**Gender** (0 *woman*, 1 *man*)	0.78***	[0.36 to 1.19]	.029	0.52*	[0.10 to 0.95]	.012	0.88***	[0.44 to 1.32]	.033	0.62***	[0.25 to 1.00]	.020	0.50*	[0.06 to 0.95]	.010
**Witnessing** Sexual Misconduct [a]	-3.43***	[-4.36 to -2.51]	.106	-3.47***	[-4.43 to -2.51]	.095	-2.78***	[-3.75 to -1.82]	.067	-2.87***	[-3.75 to -1.99]	.073	-2.86***	[-3.82 to -1.89]	.063
**Being a Target** of Sexual Misconduct [a]	1.65*	[0.31 to 2.99]	.013	1.82*	[0.34 to 3.29]	.012	0.89	[-0.54 to 2.31]	.003	-0.30	[-1.61 to 1.01]	.000	0.41	[-1.34 to 2.16]	.000
*Gender * Witness*	1.09	[-1.12 to 3.29]	.002	1.04	[-1.25 to 3.33]	.002	-0.54	[-2.79 to 1.70]	.001	-0.35	[-2.43 to 1.73]	.000	-0.32	[-2.53 to 1.88]	.000
*Gender * Target*	-0.55	[-3.27 to 2.18]	.000	-0.77	[-3.72 to 2.18]	.001	1.85	[-0.94 to 4.64]	.004	0.96	[-1.65 to 3.57]	.001	2.34	[-0.82 to 5.50]	.004
*Witness * Target*	-0.05	[-1.07 to 0.97]	.000	0.52	[-0.55 to 1.59]	.002	0.08	[-1.00 to 1.16]	.000	0.99*	[0.01 to 1.97]	.008	1.24*	[0.01 to 2.46]	.008
*Gender * Witness * Target*	-1.05	[-2.67 to 0.56]	.004	-0.37	[-2.04 to 1.31]	.000	-1.58	[-3.30 to 0.15]	.007	-0.45	[-1.99 to 1.10]	.001	-0.37	[-2.26 to 1.53]	.000
Age	0.18**	[0.06 to 0.29]	.020	0.22***	[0.11 to 0.34]	.029	0.26***	[0.13 to 0.39]	.035	0.17**	[0.06 to 0.27]	.018	0.13*	[0.01 to 0.25]	.009
Grade	-0.12	[-0.25 to 0.02]	.007	0.01	[-0.12 to 0.15]	.000	-0.15	[-0.31 to 0.00]	.008	-0.13*	[-0.25 to -0.01]	.008	-0.08	[-0.23 to 0.07]	.002

*** $p \leq .001$; ** $p \leq .01$; * $p \leq .05$; As shown, unweighted and weighted analyses yielded highly consistent results. As in primary analyses, weights were generated using population data for the NHS-E surgical workforce to produce a sample for analyses that mirrored the surgical population by gender, as well as grade and key subspecialties.

Analyses included respondents on doctor grades only. This preserved the linear interpretability of this covariate without drawing assumed equivalences across doctor, dental and nursing (or other) grades. Predictors (listed in bold text) were mean centred; [a] Represents the mean of the sexual harassment, sexual assault and rape composite scores.

Supplementary Appendix

Participant Information Sheet and Consent

Below is the verbatim wording of the information sheet and consent form:

Information Sheet

Thank you for your interest in participating in this survey. It is part of a collaborative project between the Working Party on Sexual Misconduct in Surgery and the University of Exeter. It is supported by NHS England and Improvement's Safeguarding team, the Royal College of Surgeons of England, and the British Orthopaedic Trainees' Association.

It has received HRA / HCRW Approval (Health Research Authority / Health and Care Research Wales), and ethical clearance from the University of Exeter Psychology Research Ethics Committee.

This survey aims to better understand individuals' experiences working in the healthcare profession. This includes the experiences of doctors, surgeons, dentists, nurses, medical students, dental students, nursing students and other allied healthcare professionals. This is an important time to find out what is happening in the profession, and to use the results in an effective way to ensure safe and healthy working environments.

For this survey, it is important to hear from as many members of the healthcare profession as possible. Even if some of the questions do not seem relevant to you, we would really appreciate your responses. This is important for getting a clear picture of what's happening in our profession, and we would really appreciate your input.

The survey consists of an online questionnaire that takes approximately 10-30 minutes to complete, depending on how much information you have or wish to share. For data protection purposes, the survey must be completed in one, single session (you cannot save it and return later).

You will be asked to provide general information about your background (e.g., age, gender) and your experiences at work. Please note that you will also be asked questions about sexual harassment, sexual assault, and rape in the workplace. You may leave questions unanswered if you prefer (or, where relevant, select 'prefer not to say').

In this survey, you will not be asked to provide any information that makes you identifiable. The survey includes opportunities to provide additional information about your workplace experiences in open-ended text boxes. These questions are entirely optional, and do not require you to provide any identifying information. In fact, we request that you do not provide any identifying information about yourself or others when responding to these questions, or any other questions in the survey. Should you provide any potentially identifiable information in your responses, it will be anonymised by the researchers. Where necessary, the handling of sensitive information provided via open-text boxes will be done in consultation with the University of Exeter Research Ethics and Governance team.

In 2018 regulatory changes in the way that data is processed came into force, with the EU General Data Protection Regulation 2018 (GDPR) and the Data Protection Act 2018 (DPA 2018). Since the UK left the EU, the key principles of EU GDPR have been adopted in the UK GDPR (a 'UK-only' version) and the DPA 2018 still applies.

Everyone involved in this study will keep your data safe and secure. We will also follow all privacy rules.

Specifically, all data collected in this survey will be anonymous (or anonymised, if necessary) and stored securely in line with UK GDPR and DPA 2018 for at least 5 years on a password-protected, university-affiliated cloud server (OneDrive). It will only be accessible to the principal investigators for research purposes.

As an additional safety measure, any open text / data provided within large, open-text boxes – labelled in the survey as "Open-Text Response" (does not include multiple choice questions with a short line for open text tied to a 'Not Listed' or 'Other' option) – will be stored securely in line with UK GDPR and DPA 2018 for a limited time (approximately 3 years) on a password-protected, university-affiliated cloud server (OneDrive). These responses will be stored in a file separated from all other data (only linked to the other data you provide through a randomly generated participant number) and will only be accessible to the principal investigators. In other words, you will be allocated a unique participant number to ensure your information will be protected and cannot be identified outside of the research team. Your open-text responses will be stored separately and securely from other information obtained from the research and will only be kept for a limited time (approximately 3 years) and securely destroyed (near August 2025).

This survey will help us better understand individuals' experiences in the healthcare profession. The results will be reported in scientific papers and reports, and anonymised data may be made available to others for scientific purposes. In doing so, no information will be reported or provided to others that could make you identifiable. You will remain anonymous at all times. In other words, we will make sure no one can work out who you are from the reports we write.

Your participation is voluntary, and you can withdraw from participation at any time, without giving a reason and without consequence, simply by closing out of the survey in your internet browser. Although we appreciate it if you can answer as many questions as possible, you can also leave questions unanswered. Because this survey does not request nor endeavour to receive any personally identifying information, it will not be possible to retroactively withdraw your consent, as there is no way to locate your individual responses within the file of aggregated, anonymous data (containing all respondent data). Therefore, you can stop being part of this study at any time, without giving a reason, but we will not be able to locate nor remove any information that you have already provided. This also means that we won't be able to let you see or change the data we hold about you, because there is no way to identify your data from the data of other participants.

If any questions in this survey cause you distress or discomfort, please contact RCS England's Confidential Support and Advice Service (0800 028 0199; confidential support and advice from a trained counsellor, available 24/7; state your organisation as "Royal College of Surgeons;" [Link to RCS-Eng advice and support site]). You can also contact the BMA (0330 123 1245; Free, confidential, 24/7 counselling and peer support services open to all doctors and medical students regardless of BMA membership; state "BMA" as your organisation; [Link to BMA advice and support site]).

You can also contact your GP to discuss matters relating to your mental health and well-being. Your GP can also provide more information about relevant services and supports that you can access. There are other services as well that can help anyone who has been sexually assaulted, raped or abused (for more information: Link to NHS advice and support site). Free legal advice is

also available to women in England and Wales experiencing sexual harassment at work (020 7490 0152; Link to RightsofWomen advice and support site).

Please do not hesitate to contact us by email (C.T. Begeny; C.Begeny@exeter.ac.uk) if you require additional information or wish to provide any comments. If you have any ethical concerns, you may contact the Chair of the Committee for Ethics in Psychology at the University of Exeter, Dr. ****** (******@exeter.ac.uk).

The University of Exeter terms its lawful basis to process personal data for the purposes of carrying out research as being in the 'public interest.' The University continues to be transparent about its processing of your personal data and the participant information sheet should provide a clear explanation of how your data will be collected, processed, stored and destroyed. If you have any queries about the University's processing of your personal data that cannot be resolved by the research team, further information can be obtained from the University of Exeter's Data Protection Officer via the web-link: https://www.exeter.ac.uk/aboutoursite/dataprotection/dpo/.

If you have any concerns about how your data is controlled and managed for this study, please contact the Sponsor Representative:

Ms Pam Baxter - Research Governance Manager (Health & Social Care)
University of Exeter, Research Ethics and Governance Office, Lafrowda House, St Germans Road Exeter EX4 6TL / Tel: ****** / Email: ******

Consent
To proceed to the survey, **please click on ALL statements below** to confirm your acknowledgement of them, and your consent to participate. If you do not wish to participate, you can simply close out of the survey (close this window in your internet browser).

- ▢ I confirm that I have read the information sheet (above) for this survey. I have had the opportunity to consider the information.
- ▢ I understand that my participation is voluntary and that I am free to withdraw at any time without giving any reason and without my legal rights being affected.
- ▢ I understand that relevant sections of the data collected during this survey will be looked at by members of the research team, where it is relevant to my taking part in this survey. I give permission for these individuals to access the data that I provide.
- ▢ I am 18 or older, and **I agree to take part in this survey**.
- ▢ This project has been reviewed and approved by the University of Exeter Psychology Research Ethics Committee. I have read the preceding statement and **I consent to take part in this survey**. If you do not consent to take part in this survey, do not click on this statement; instead, please simply close out of the survey (close this window in your internet browser).

Debriefing Form

Below is the verbatim wording of the debriefing form:

Debriefing Form
Thank you for participating in this survey. Your participation is extremely valuable to us and will contribute to improving our understanding of individuals' experiences working in the healthcare

profession, including with sexual harassment, assault and rape. In affiliation with the University of Exeter, and with the support of the NHS England and Improvement's Safeguarding team, the Royal College of Surgeons of England, and the British Orthopaedic Trainees' Association, we are carrying out this research in efforts to better understand and improve the experiences and working environments for those facing sexual misconduct in healthcare settings. We thank you for your time and honest responses in this endeavour.

This survey did not entail any experimental manipulations or participant deception.

If any questions in this survey caused you distress or discomfort, please contact RCS England's Confidential Support and Advice Service (0800 028 0199; confidential support and advice from a trained counsellor, available 24/7; state your organisation as "Royal College of Surgeons;" [Link to RCS-Eng advice and support site]). You can also contact the BMA (0330 123 1245; Free, confidential, 24/7 counselling and peer support services open to all doctors and medical students regardless of BMA membership; state "BMA" as your organisation; [Link to BMA advice and support site]).

You can also contact your GP to discuss matters relating to your mental health and well-being. Your GP can also provide more information about relevant services and supports that you can access. There are other services as well that can help anyone who has been sexually assaulted, raped or abused (for more information: Link to NHS advice and support site). Free legal advice is also available to women in England and Wales experiencing sexual harassment at work (020 7490 0152; Link to RightsofWomen advice and support site).

Because this survey does not request nor endeavour to receive any personally identifying information, it will not be possible to retroactively withdraw your consent, as there is no way to locate your individual responses within the file of aggregated, anonymous data (containing all respondent data). This also means that we won't be able to let you see or change the data we hold about you, because there is no way to identify your data from the data of other participants.

Please do not hesitate to contact us by email (C.T. Begeny; C.Begeny@exeter.ac.uk) if you require additional information or wish to provide any comments. If you have any ethical concerns, you may contact the Chair of the Committee for Ethics in Psychology at the University of Exeter, Dr. ****** (******@exeter.ac.uk).

The University of Exeter terms its lawful basis to process personal data for the purposes of carrying out research as being in the 'public interest.' The University continues to be transparent about its processing of your personal data and the participant information sheet should provide a clear explanation of how your data will be collected, processed, stored and destroyed. If you have any queries about the University's processing of your personal data that cannot be resolved by the research team, further information can be obtained from the University of Exeter's Data Protection Officer via the web-link:
https://www.exeter.ac.uk/aboutoursite/dataprotection/dpo/.

If you have any concerns about how your data is controlled and managed for this study, please contact the Sponsor Representative:
Ms Pam Baxter - Research Governance Manager (Health & Social Care) University of Exeter, Research Ethics and Governance Office, Lafrowda House, St Germans Road Exeter EX4 6TL / Tel: ****** / Email: ******

Supplementary Tables

Tables S1 - S3

See pg. 6 for *Table S1. Items assessing frequency of witnessing sexual harassment, sexual assault and rape among colleagues*.

See pg. 8 for *Table S2. Items assessing frequency of being a target of sexual harassment, sexual assault and rape among colleagues*.

See pg. 13 for *Table S3. Evaluations of organisations' handling of sexual harassment and assault as a function of one's experiences with sexual misconduct*.

Table S4. List of key organisations that provided support (non-financial) for the study

Organisations Providing Supportive Endorsement of Study
NHS England Safeguarding
Royal College of Surgeons of England
British Orthopaedic Trainees Association
Royal College of Surgeons of Edinburgh
Royal College of Physicians and Surgeons of Glasgow
British Orthopaedic Association
British Association of Oral and Maxillofacial Surgeons
Association of Surgeons in Training
Society of Women in Oral and Maxillofacial Surgery
Royal College of Anaesthetists
Association for Perioperative Practice
**Organisations Providing Support with Participant Recruitment**
Health Education England
Royal College of Surgeons of England
British Orthopaedic Trainees Association
Royal College of Physicians and Surgeons of Glasgow
British Orthopaedic Association
British Association of Oral and Maxillofacial Surgeons
Association of Surgeons in Training
Society of Women in Oral and Maxillofacial Surgery
Royal College of Anaesthetists
Association for Perioperative Practice
UK Committee of Postgraduate Dental Deans and Directors
University College London Women in Surgery
Barts Health NHS Trust

NHS: National Health Service; Organisations approached for support (support not provided): Royal College of Nursing, Royal College of Surgeons of Ireland, Association of Surgeons of Great Britain and Ireland

References

1 Fitzgerald LF, Gelfand MJ, Drasgow F. Measuring sexual harassment: Theoretical and psychometric advances. *Basic Appl Soc Psychol*. 1995; **17**: 425–445.

2 Fitzgerald LF, Shullman SL, Bailey N, Richards M, Swecker J, Gold Y, *et al.* The incidence and dimensions of sexual harassment in academia and the workplace. *J Vocat Behav*. 1988; **32**: 152–175.

3 Fitzgerald LF, Drasgow F, Hulin CL, Gelfand MJ, Magley VJ. Antecedents and consequences of sexual harassment in organizations: A test of an integrated model. *J Appl Psychol*. 1997; **82**: 578–589.

4 Sexual Offences Act 2003. Available from: https://www.legislation.gov.uk/ukpga/2003/42/section/1

5 UK Ministry of Justice. Code of Practice for Victims of Crime in England and Wales (Victim's Code). Available from: https://www.gov.uk/government/publications/the-code-of-practice-for-victims-of-crime/code-of-practice-for-victims-of-crime-in-england-and-wales-victims-code

6 ACAS. Sexual harassment: The Advisory, Conciliation and Arbitration Service. Available from: https://www.acas.org.uk/sexual-harassment

7 National Occupational Standards. SFJSV1: Advocate on behalf of victims/survivors of sexual violence. Available from: https://www.ukstandards.org.uk/PublishedNos-old/SFJSV1.pdf

8 Citizens Advice. Check what you can do about harassment. Available from: http://www.citizensadvice.org.uk/law-and-courts/discrimination/taking-action-about-discrimination/taking-action-about-harassment/

9 RAINN. Key Terms and Phrases. Available from: https://www.rainn.org/articles/key-terms-and-phrases

10 NHS Digital. NHS Hospital and Community Health Service workforce statistics: HCHS doctors in surgical group specialties, by specialty, grade and gender. 2022. Available from: https://digital.nhs.uk/supplementary-information/2022/hchs-doctors-in-selected-surgical-specialties-by-gender-and-grade-jun-22
